# A novel mutation in the *VHL* gene in a Chinese family with von Hippel-Lindau disease

**DOI:** 10.1186/s12881-018-0716-4

**Published:** 2018-11-26

**Authors:** Xing Wu, Lanlan Chen, Yixin Zhang, Hainan Xie, Meirong Xue, Yi Wang, Houbin Huang

**Affiliations:** 1 Department of Ophthalmology, Chinese PLA General Hospital, Beijing, 100853 China; 2 Department of Ophthalmology, Hainan Hospital of Chinese PLA General Hospital, Sanya, 572013 Hainan Province China

**Keywords:** VHL, Missense mutation, W117R

## Abstract

**Background:**

Von Hippel-Lindau (VHL) disease is an autosomal dominant inherited cancer syndrome, and *VHL* is identified as a tumor suppressor gene. The main objective of this study was to identify disease-causing mutations in a Chinese family affected with VHL disease.

**Methods:**

Genomic DNA was extracted from peripheral blood from a Chinese family with VHL. A predicted pathogenic variant was identified by targeted exome capture technology and next-generation sequencing.

**Results:**

A novel heterozygous mutation (c.349 T > A, p.W117R) was detected in affected family members. No mutation was detected in unaffected family members or in the 150 normal controls. The mutation segregated with the disease phenotype throughout three generations. Histopathological examination revealed the characteristics of hemangioblastoma.

**Conclusions:**

A novel W117R was detected in the *VHL* gene that caused retinal hemangioblastomas in affected members of a Chinese family.

## Introduction

VHL disease is an autosomal dominant inherited cancer syndrome, including brain and retinal hemangioblastomas, spinal hemangioblastomas, renal carcinomas, pancreatic cancers, and pheochromocytomas. *VHL* is identified as a tumor suppressor gene, which has three exons encoding the VHL protein (pVHL). Germline mutation of the *VHL* gene is the leading cause of the disease. *VHL* gene is highly conserved across species and is located on the short arm of chromosome 3, 3p25-26 [1]. The mutant allele in most VHL patients is inherited from an affected parent. Missense mutations and nonsense mutations are the most common. Somatic mutation cases with homozygous inactivation of the *VHL* alleles are tumorigenic [2]. The incidence of the disease is about 1/36000 live births per year with high penetrance (> 90%) before 65 years of age [1, 3]. Of all VHL patients, up to 85% develop retinal hemangioblastomas, whereas tractional retinal detachment with proliferated epiretinal membranes (ERM) is detected in 9% patients [4]. It is very difficult to treat optic nerve hemangioblastomas, which usually leads to poor visual prognosis.

Mutations in the *VHL* gene inactivate the VHL protein and induces excessive vascular endothelial growth factor (VEGF), which promotes blood vessels to proliferate and to form retina hemangioblastomas in nonhypoxic conditions [4]. Many VHL patients, commonly younger than 50 years of age without routine screening surveys, die of renal cell carcinomas and central nervous system hemangioblastomas [5]. Retinal hemangioblastomas, usually noted in the temporal peripheral region of the retina, are often the earliest presentation of VHL patients in their mid-twenties [6].

Herein, we summarize the clinical, histological and genetic data of a VHL Chinese family and identify a novel heterozygous mutation in the *VHL* gene.

## Materials and methods

### Patients recruitment and evaluation

**Fig. 1:**
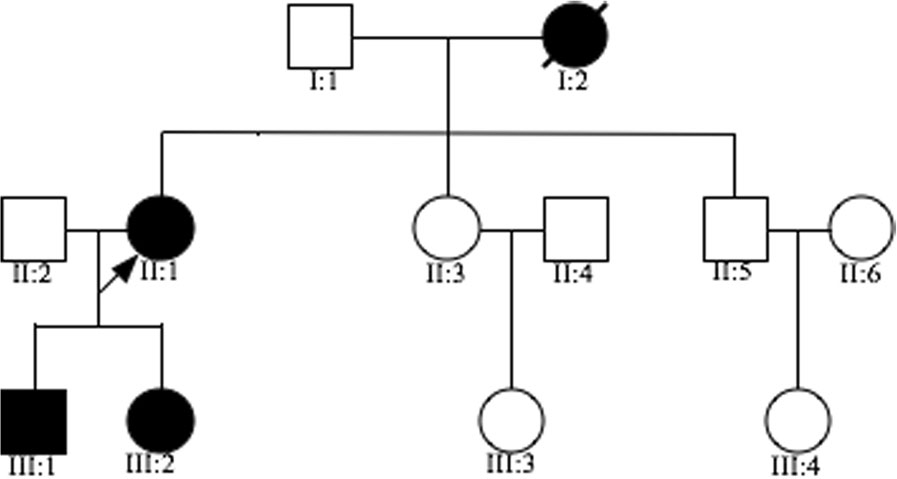
The pedigree of the Chinese family. The filled symbols represent affected individuals and unfilled symbols unaffected individuals. Squares signify men, and circles women. An arrow indicates the proband

A Chinese family from southern China showed an autosomal dominant inheritance pattern for VHL (Fig. 1) that affected 3 of the 7 living family members. Medical and ophthalmic histories were obtained, and ophthalmological examinations were performed. All procedures were performed according to the guidelines of the Declaration of Helsinki for research involving human subjects, and were approved by the Ethics Committee of Chinese PLA General Hospital. Informed written consent comprised DNA extraction, gene analysis and ophthalmological examinations. 150 normal controls were used in the study. Written informed consent was obtained from all participants, while the consent of the 6-year-old and 12-year-old patients was signed by their father.

### Targeted capture preparation, sequencing and validation

Genomic DNA was extracted from peripheral blood using a DNA extraction kit (TIANGEN, Beijing, China) according to the manufacturer’s instructions. DNA was purified and quantified using a Nanodrop 2000 spectrophotometer (Thermal Fisher Scientific, DE). DNA fragments with sizes ranging from 350 bp to 450 bp and those including the adapter sequences were selected for the DNA libraries.

The coding regions of 381 genes associated with retinal diseases were captured using the GenCap custom enrichment kit (MyGenostics, Beijing, China). Genomic DNA was fragmented and mixed with GenCap probe (MyGenostics, Beijing) for PCR and hybridization as previously described [7]. PCR products were purified using SPRI beads (Beckman Coulter, Brea, CA, USA), according to the protocol.

The enriched libraries were sequenced using an Illumina Nextseq 500 sequencer (Illumina, San Diego, CA, USA) for paired-end reads of 150 bp. All mutations identified by Nextseq 500 sequencing were confirmed by PCR. The DNA samples were amplified with the forward primer 5′- CTGACCTCATGATCCGCCT-3′ and the reverse primer 5′- GGTCTATCCTGTACTTACCACAA-3′ (292 bp, VHL exon2 chr3:10188086-10188378). Genomic DNA from all available family members were obtained for PCR and next-generation sequencing (NGS). Sites of variation were identified through a comparison of DNA sequences with the corresponding GenBank (www.ncbi.nlm.nih.gov) reference sequences.

### Informatics analysis

Following sequencing, raw image files were processed using Bcl2Fastq software(Bcl2Fastq 2.18.0.12, Illumina, Inc.) for base calling and raw data generation. Low-quality variations were filtered out using a quality score ≥ 20. Short Oligonucleotide Analysis Package (SOAP) aligner software (SOAP2.21; soap.genomics.org.cn/soapsnp.html) was then used to align the clean reads to the reference human genome (hg19). Polymerase chain reaction (PCR) duplicates were removed using the Picard program. Subsequently, single nucleotide polymorphisms (SNPs) were determined using the SOAPsnp program, reads were realigned using Burrows-Wheeler Aligner software 0.7.15, and the deletions and insertions (InDels) were detected using Genome Analysis Toolkit software 3.7. The identified SNPs and InDels were annotated using the Exome-assistant program (https://bmcgenomics.biomedcentral.com/articles/10.1186/1471-2164-13-692). MagicViewer was used to view the short read alignment, and confirm the candidate SNPs and InDels. Non-synonymous variants were evaluated using the four algorithms, PolyPhen (http://genetics.bwh.harvard.edu/pph2/), Sorting Intolerant From Tolerant [SIFT; (http://sift.jcvi.org/)], Protein Analysis Through Evolutionary Relationships (PANTHER; www.pantherdb.org) and Pathogenic Mutation Prediction (Pmut; http://mmb.pcb.ub.es/PMut/) to predict pathogenicity. The Human Gene Mutation Database (HGMD) is used to confirm the novelty of the variant (http://www.hgmd.cf.ac.uk/ac/index.php).

## Results

### Phenotyping

#### Case II:1 (the proband)

**Fig. 2:**
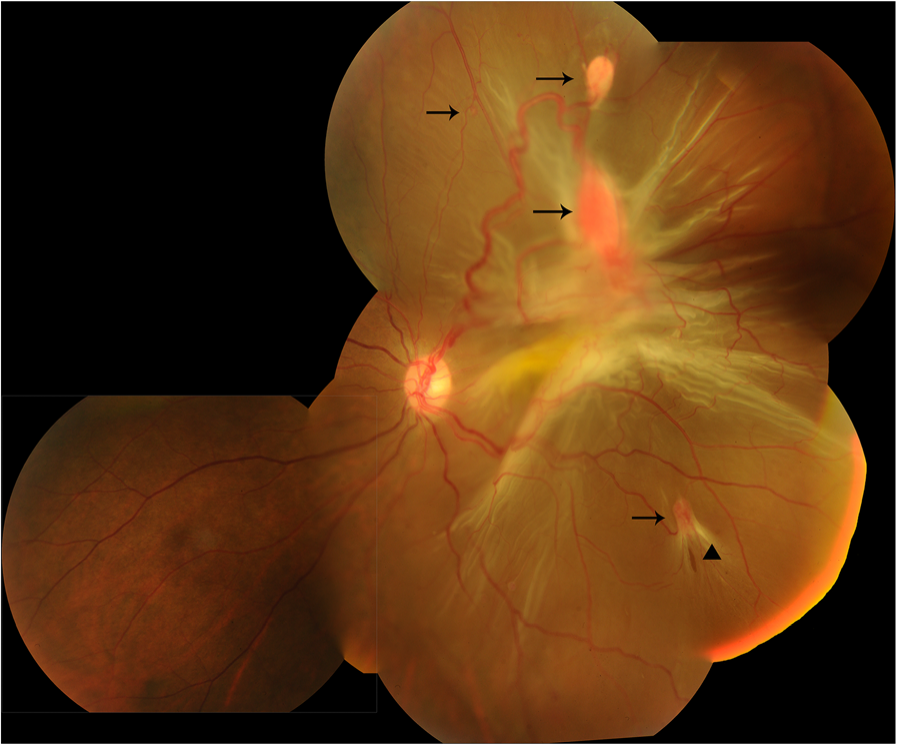
Fundus examination of the proband’s left eye. Arrows showed several retinal hemangioblastomas with dilated vessels, ranging from small lesions to large ones, the largest being 1.5 papilla diameters. There are apparent dense epi-retinal membranes connected to the hemangioblastoma. Tractional retinal detachment is shown from the 12-o’clock to 6-o’clock position. The arrowhead shows a small retinal tear at the base of the inferior-temporal hemangioblastoma with fibrous changes

The 32-year-old female attended the outpatient clinic of our hospital in June 2017. She complained of blurred vision for the past 2 years in her left eye. The patient had previously undergone the operation for cerebellar hemangioblastoma 3 years ago. Family history revealed that the patient’s mother had blindness in one eye and died of renal carcinoma. Upon initial examination, the patient’s BCVA was NLP OD and 0.04 OS. Her intraocular pressure was 39 mmHg OD and 17 mmHg OS. Fundus examinations showed several retinal hemangioblastomas in the temporal area of the left eye. Retinal traction detachment was noted due to the formation of proliferative membranes connected to the hemangioblastomas (Fig. 2). Because of a complicating cataract, the fundus was not visible in the right eye, and B ultrasound scan showed proliferative vitreoretinopathy. She underwent PPV, hemangioblastoma resectomy and oil temponade in the left eye. BCVA improved to 0.2 OS postoperatively.

#### Case III:1 (the proband’s son)

The asymptomatic 6-year-old son of the proband had a visual acuity of 1.0 in both eyes. Fundus examination revealed a very small hemangioblastoma in the distant peripheral retina of his right eye, while his left eye was normal.

#### Case III:2 (the proband’s daughter)

**Fig. 3:**
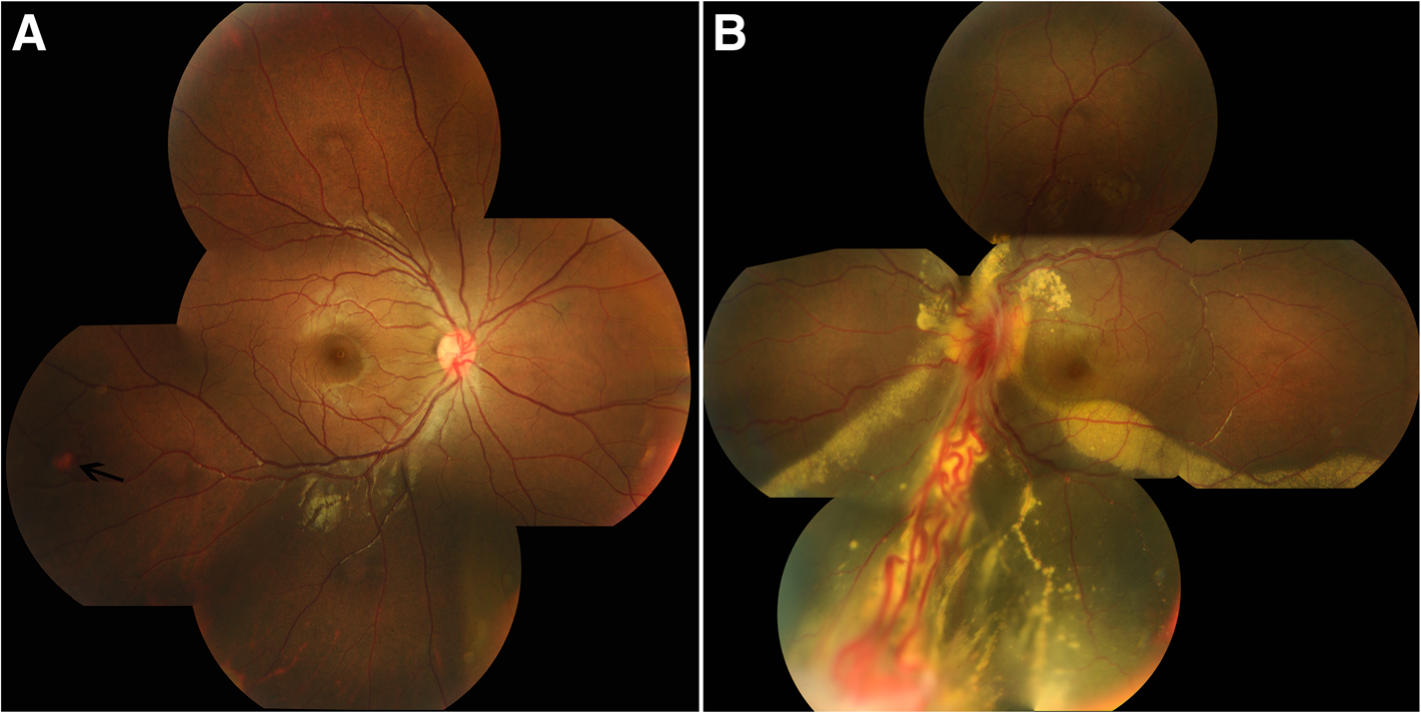
Fundus examinations of the 12-year-old daughter. Panel **a**: The arrow shows a small isolated hemangioblastoma in the infratemporal region of the retina (right eye). Panel **b**: **a** sizeable hemangioblastoma is noted in the inferior periphery of the retina, with the feeder vessels dilated with marked enlargement and tortuosity. Exudative retinal detachment with massive hard exudate deposition is seen in the inferior retina (left eye)

The 12-year-old daughter of the proband complained of a curtain coming across the vision in her left eye during the screening examination. Her BCVA was 1.0 OD and 0.8 OS. Fundus examinations revealed a small hemangioblastoma in the infratemporal region of the peripheral retina in her right eye. A sizeable hemangioblastoma with 2.5 papilla diameters and prominant feeder vessels were detected in the inferior peripheral retina of her left eye, fractional retinal detachment was demonstrated with fibrovascular proliferation (Fig. 3a-b).

#### Case I:2 (the proband’s mother)

The mother of the proband has passed away. She had blindness in one eye and died of renal carcinoma, so she was supposed to be the variant based on her medical history.

### Histopathologic analysis

Normal hematoxylin-eosin stain of the excised specimen from the proband showed abundant stromal cells packed with vascular channels. Immunohistochemically, the specimen was positive for CD31, CD34 and NSE.

### Genotyping

The coding regions of 381 gene complicated in heritable retinal disease were captured and sequenced by NGS and PCR using genomic DNA from the proband (II:1). PCR was used to confirm the variation (292 bp, VHL exon2 chr3:10188086-10,188,378). The coverage statistics are showed in Table 1. I:2 has passed away, so her gene analysis was not available. DNA sequencing revealed the 3 living affected members (II:1, III:1, III:2) in the family harbored a novel heterozygous missense mutation. The nucleotide transversion (c.349 T > A) within exon 2 in *VHL* gene resulted in a change of tryptophan to arginine (W117R). Tryptophan is non-polar aromatic, and when substituted by arginine, it can damage the stability of the protein. None of the 4 unaffected individuals (I:1, II:2, II:3, II:5) and the 150 normal controls have this mutation in *VHL* gene. The mutation consegregated with the disease phenotype. W117R variant would be classified as “likely pathogenic” (PM2, PM5, PP1, PP3, PP4) using American College of Medical Genetics and Genomics (ACMG) criteria.

**Table 1:** Coverage statistics with next-generation sequencing in the proband with VHL disease

Sample	Initial bases on target (bp)	Bases covered on target (bp)	Coverage of target region	Average sequencing depth on target	Fraction of target covered with		
					at least 4×	at least 10×	at least 20×
II:2	1285,536	1281,093	99.65%	582.51	99.41%	99.25%	98.84%

## Discussion

VHL disease mostly manifests with cerebellar or retinal hemangioblastomas. The second most common feature is renal cell carcinoma. Germline VHL mutations induce different phenotypes of cancer [8]. Truncating mutations and large deletions are most commonly associated with the phenotype of renal carcinoma, type I. Missense mutations are commonly associated with in the phenotypes of pheochromocytoma alone or accompanied with renal carcinoma, type II. Genetic testing can allow for discontinuation of surveillance for family members without VHL gene mutations. About 60% of VHL patients deny a family history, suggesting a high frequency of novel mutations in the VHL gene [9, 10].

VHL disease was identified in this family because of ocular findings and gene analysis. Retinal hemangioblastomas are an important ocular manifestation of VHL, ranging from very small capillary abnormalities to large lesions that cause tractional retinal detachment. Initially, a small hemangioblastoma presents as a red dot with a diameter of hundred microns, then develops into a large nodule with a distinctive clinical appearance [5]. Small lesions are easily to be treated by photocoagulation. Transscleral cryotherapy is used to treat the larger lesions in the extreme periphery of the retina. Large lesions (> 1.5 mm in diameter) are often difficult to treat by laser photocoagulation or cryotherapy. Vitrectomy is performed in patients with tractional retinal detachment or severe vitreous hemorrhage. Peripheral large lesions can be excised during an operation, but this approach is not appropriate for lesions adjacent to the optic disc. Optic nerve hemangioblastomas grow slowly, and visual acuity deteriorates with hard exudates, macular pucker, and epiretinal membranes.

Anti-VEGF therapeutic approaches have been developed in recent years. The increased expression of VEGF mRNA appears to correlate with retinal tumor formation [3]. In pVHL-defective cells, leads to hypoxia-inducible factor (HIF) cannot be degradated. The excessive accumulation of HIF protein hypoxia and the up-regulation of hypoxia-induced genes, including erythropoietin (Epo), VEGF, transforming growth factor (TGF), and platelet-derived growth factor (PDGF) [11]. HIF also activates NF-κB and cyclin A, producing many growth factors and cytokines, and the release epidermal growth factor (EGF) and fibroblast growth factor (FGF) [12]. Therefore, anti-VEGF alone is not sufficient to treat ocular lesions in VHL patients. The intravitreal injection of anti-VEGF drugs does not alter the size of hemangioblastoma, but decreases retinal exudates and macular edema in some patients [13–15].

The novel W117R mutation identified in the VHL gene of this Chinese family was detected in 3 affected individuals but was not detected in 4 unaffected members. These findings support the hypothesis that the W117R mutation causes VHL disease in the patients. The mutation exhibited 100% penetrance in this family, even in young children.

pVHL binds to hypoxia-inducible factor (HIF). The ubiquitination of HIF by the β-domain of amino acids pVHL (63-155) plays a key role in the cellular adaptive response to changes in oxygen availability. The hydroxyproline of HIF inserts into a gap in the pVHL hydrophobic core, which is a hotspot of tumorigenic VHL mutations [16]. The tryptophan at amino acid position 117 lies in the pVHL hydrophobic core, and plays a central role in stabilizing pVHL. In our study, tryptophan is substituted by Arginine at 117 codon, which destroys the hydrophobic core and changes the protein tertiary structure. According to previous studies of the pVHL-HIF1α complex, mutations of Trp117, Ser111, and Tyr98 of pVHL interfere with binding of HIF-1α to pVHL [17]. Therefore, the W117R mutation is expected to abolish the ability of pVHL to bind HIF, is the leading cause of VHL disease in this Chinese family.

## Conclusions

In conclusion, we have identified a novel heterozygous mutation in the VHL gene (c.349 T > A, p.W117R) of a Chinese family, which broadens the spectrum of VHL gene mutations in Chinese patients.

## Abbreviations

VHL: Von Hippel-Lindau
pVHL: VHL protein
HIF: hypoxia-inducible factor
